# The “completely randomised” and the “randomised block” are the only experimental designs suitable for widespread use in pre-clinical research

**DOI:** 10.1038/s41598-020-74538-3

**Published:** 2020-10-16

**Authors:** Michael F. W. Festing

**Affiliations:** c/o The Medical Research Council, 2nd. floor, David Phillips Building, Polaris House, North Star Av., Swindon, Wiltshire SN2 1FL UK

**Keywords:** Experimental models of disease, Medical research

## Abstract

Too many pre-clinical experiments are giving results which cannot be reproduced. This may be because the experiments are incorrectly designed. In “Completely randomized” (CR) and “Randomised block” (RB) experimental designs, both the *assignment of treatments* to experimental subjects and the *order* in which the experiment is done, are randomly determined. These designs have been used successfully in agricultural and industrial research and in clinical trials for nearly a century without excessive levels of irreproducibility. They must also be used in pre-clinical research if the excessive level of irreproducibility is to be eliminated. A survey of 100 papers involving mice and rats was used to determine whether scientists had used the CR or RB designs. The papers were assigned to three categories “Design acceptable”, “Randomised to treatment groups”, so of doubtful validity, or “Room for improvement”. Only 32 ± 4.7% of the papers fell into the first group, although none of them actually named either the CR or RB design. If the current high level of irreproducibility is to be eliminated, it is essential that scientists engaged in pre-clinical research use “Completely randomised” (CR), “Randomised block” (RB), or one of the more specialised *named* experimental designs described in textbooks on the subject.

## Introduction

Excessive numbers of randomised, controlled, pre-clinical experiments give results which can’t be reproduced^1,2^. This leads to a waste of scientific resources with excessive numbers of laboratory animals being subjected to pain and distress^3^. There is a considerable body of literature on its possible causes^4–7^, but failure by scientists to use named experimental designs described in textbooks needs further discussion.

**Figure 1:**
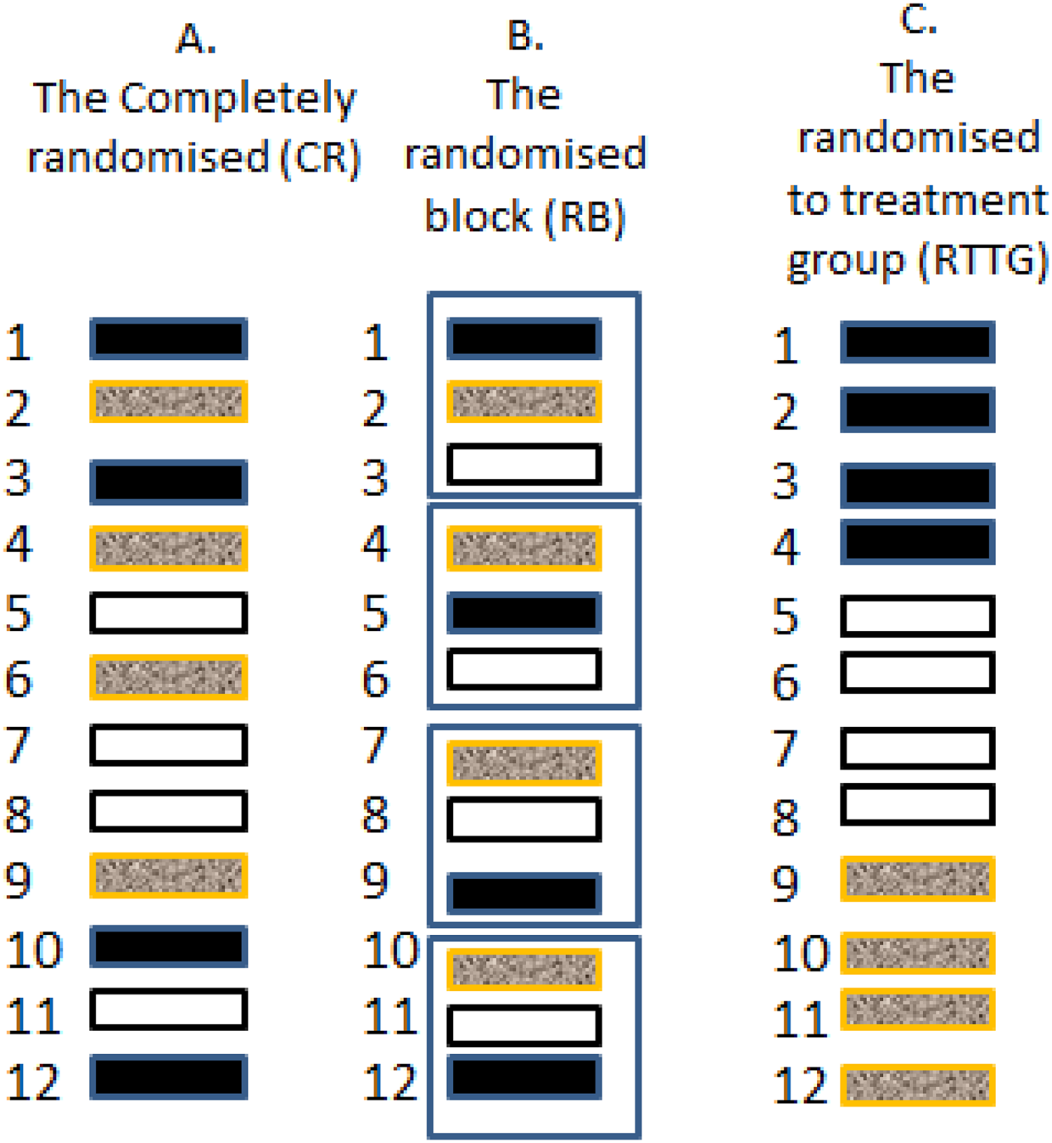
Representation of three experimental designs, each with three treatments (colours) with a sample size of four (for illustration). Each small rectangle represents an experimental unit (for example, a single animal in a cage). Designs A and B can have any number of treatments and sample sizes as, well as additional factors such as both sexes, or more than one strain. Design C is not statistically valid. (**A**) The “Completely randomised” (CR) design. Both assignment of treatments to subjects, and the order in which the experiment is done are randomly determined. This design can accommodate unequal sample sizes. Randomisation was done using EXCEL: Four “A”s, four “B”s and four “C”s were entered into column one and 12 random numbers were put in column two using the command “ = rand()”, and pulling down on the small box on the lower right of the cell. Columns one and two were then marked and sorted on column two using “data, sort”. The row numbers represent individual identification numbers. Different results will be obtained each time. (**B**) The “Randomised block” (RB) design. In this example the experiment has four blocks (outer rectangles) each having a single individual receiving each of the three treatments, in random order. The blocks can be separated in time and/or location. Randomisation was done as follows: Four “A”s, “B”s and “C”s, were put in column 1 and the numbers 1–4 repeated three times were put in column 2. Twelve random numbers were then put in column three, as above. All three columns were then marked and sorted first on column two and then on column three. Row numbers are the individual identity numbers. (**C**) The “Randomisation to treatment group” (RTTG) “design”. *This is not a valid design* because treatment and environmental effects are confounded. Any environmental effect that differs between groups may be mistaken for the effects of the treatment, leading to bias and irreproducible results.

Only two designs are suitable for widespread use in pre-clinical research: “Completely randomised” (CR) shown in Fig. 1A, and “Randomised block” (RB) shown in Fig. 1B. In the CR design, each subject (experimental unit) has one of the treatments randomly assigned to it, so that subjects receiving different treatments are *randomly intermingled* within the research environment. Results can be statistically analysed using a one-way analysis of variance, with the usual assumptions of homogeneity of variances and normality of the residuals.

In the *RB* design, the experiment is split up into a number of independent “blocks” each of which has a single subject assigned at random to each treatment. When there are only two treatments, this is known as a “matched pairs” design. The whole experiment consists of “N” such blocks where N is sample size. A two-way analysis of variance without interaction is used to analyse the results. The matched pairs design can also be analysed using a one-sample t-test.

Unfortunately, most authors appear to use the *in-valid* “Randomisation to treatment group” (RTTG) design, shown in Fig. 1C. In this design, subjects are randomly assigned to physical treatment groups but the *order* in which the experiment is done is not randomised. This is not valid because each treatment group will occupy a different micro-environment, the effects of which may be mistaken for treatment effects, leading to bias and irreproduciblity.

## The origin of randomized controlled experiments

Randomized controlled experiments have a long history of successful use in agricultural research. They were developed largely by R. A. Fisher in the 1920s as a way of detecting small but important differences in yield of agricultural crop varieties or following different fertilizer treatments^8^. Each variety was sown in several adjacent field plots, chosen at random, so that variation among plots growing the same and different crop varieties could be estimated. He used the analysis of variance, which he had invented in previous genetic studies, to statistically evaluate the results.

Fisher noted that in any experiment there are *two* sources of variation which need to be taken into account if true treatment differences are to be reliably detected. First, is the variation among the experimental subjects, due for example, to the number of grains in a given weight of seed, or to individual variation in a group of mice. Second is the variation caused during the course of the experiment by the research environment and in the assessment of the results. *B*o*th* types of variation must be controlled if bias and irreproducibility are to be avoided.

In most pre-clinical research the inter-individual variation can be minimised by careful selection of experimental subjects. But variation associated with the environment caused, for example, by cage location, lighting levels, noise, time of day and changes in the skill of investigators must also be considered. Fisher’s designs minimised bias by using uniform material and by *replication* and *randomisation* so that plots receiving different varieties were randomly “intermingled” in the research environment.

According to Montgomery^9^ *“By randomization we mean that both the allocation of the experimental material, and the order in which the individual runs or trials of the experiment are to be performed, are randomly determined”.*

The RB design often provides better control of both inter-individual and environmental variation. Subjects within a block can be matched and each block has a small environmental footprint, compared with the CR design. In one example this resulted in extra power equivalent to using about 40% more animals^10^. The RB design is also convenient because individual blocks can be set up over a period of time to suit the investigator. Positive results will only be detected if the blocks give similar results, as assessed by statistical analysis^11,12^. Montgomery^9^^,p 12^ even suggests that blocking is one of the three basic principles of experimental design, along with “replication” and “randomisation”.

Fisher and others invented a few other named designs including the “Split plot”, the “Latin square” and the “Cross-over” designs. These can also be used in pre-clinical research in appropriate situations^13^, although they are not discussed here.

## The research environment is an important source of variation in pre-clinical research

In most pre-clinical experiments inter-individual variation can be minimised by choosing animals which are similar in age and/or weight. They will have been maintained in the same animal house and should be free of infectious disease. They may also be genetically identical if an inbred strain is used. So the *research environment* may be the main source of inter-individual variation.

Temporal variation due to circadian and other rhythms such as cage cleaning and feeding routines can affect the physiology and behaviour of the animals over short periods, as do physical factors such as cage location, lighting and noise^14^. If two or more animals are housed in the same cage they will interact, this can increase physiological variation. Even external factors such as barometric pressure can affect the activity of mice^15^. Staff may also become more proficient at handling animals, applying treatments, doing autopsies and measuring results during the course of an experiment, leading to changes in the quality of data.

To avoid bias, cages receiving different treatments must be *intermingled* (see Fig. 1A,B), and results should be assessed “blind” and in random order. This happens automatically if subjects are only identified by their identification number once the treatments have been given.

The RB design, is already widely used in studies involving pre-weaned mice and rats^11^. No litter is large enough to make up a whole experiment. So each is regarded as a “block” and one of the treatments, chosen at random, is assigned to each pup within the litter. Results from several litters are then combined in the analysis^16^.

## Possible confusion associated with the meaning of the word “group”

Research scientists are sometimes urged to “randomise their subjects to treatment groups”. Such advice is ambiguous. According to Chambers Twentieth Century Dictionary (1972) the word “*group*” can mean *“a number of persons or things together”* or *“a number of individual things related in some definite way differentiating them from others”*.

Statisticians involved in clinical trials sometimes write about “randomising patients to treatment groups”. Clearly, they are using the second definition as there are no physical groups in a clinical trial. But if scientists assign their animals to physical groups (“….things together”), they will be using the invalid “Randomisation to treatment group” (RTTG) design shown in Fig. 1C, possibly leading irreproducibility.

## A sample survey of experimental design in published pre-clinical papers

A survey of published papers using mice or rats was used to assess the use of CR, RB, or other named experimental designs. PubMed Central is a collection of several million full-text pre-clinical scientific papers that can be searched for specific English words. A search for “Mouse” and “Experiment” retrieved 682,264 papers. The first fifty of these had been published between 2014 and 2020. They were not in any obvious identification number or date order. For example, the first ten papers had been published in 2017, 17, 19, 19, 19, 18, 15, 16, 19, and 18. And the first two digits of their identification numbers were 55, 55, 66, 65, 66, 59, 71, 61, 46 and 48. In order to introduce a random element to the selection, only papers with an even identification number were used.

Each paper was searched for the words “random”, “experiment”, “statistical”, “matched” and other words necessary to understand how the experiments had been designed. Tables and figures were also inspected. The discipline and type of animals which had been used (wild-type, mutant, or genetically modified) was also noted. The aim was to assess the design of the experiments, not the quality of research.

Most papers involved several experiments, but the designs were usually similar. All were assessed and re-assessed, blind to the previous scores, after an interval of approximately 2 weeks. The results in seventeen of the papers were discordant so they were reassessed.

## Papers which used laboratory mice

The results for mice and rats are summarised in Table 1. Thirty six (72 ± 3.2%) of the “mouse” papers involved genetically modified or mutant mice. Each was assigned to one of three categories:

1. “Apparently well designed” (13 papers, 26 ± 1.6%). None of these papers mentioned either the CR or RB design by name, although a few of them appeared to have used one of these designs. For example, one stated: "All three genotypes were tested on the same day in randomized order by two investigators who were blind to the genotypes." This was scored as a CR design.
2. “Room for improvement” (22 papers, 44 ± 3.5%). None of these papers used the word “random” with respect to the assignment of treatments to the animals, or the order in which the experiment was done, although it was sometimes used in other contexts. So these papers had not, apparently, used any named experimental design, so were susceptible to bias.
3. “Randomised to group” (15, papers, 30 ± 2.1%). These papers stated that the subjects had been “Randomised to the treatment groups”. The most likely interpretation is that these were physical groups, so the experiments had used the statistically invalid RTTG design as shown in Fig. 1C. However, as noted above, the word group is ambiguous. if it meant that one of the treatments, chosen at random, had been assigned to each animal, then this would have constituted a “Completely randomised” (CR) design. As the first interpretation seems to be most likely, these experiments were classified as being of doubtful validity.

**Table 1:** Summary of the results of the survey. Percent and 95% confidence interval.

Category	Mice (n = 50)	Rats (n = 50)	Both species (n = 100)
“Design acceptable”	26.0, (14.6–40.3)	38.0, (24.5–52.8)	32.0, (23.0–42.0)
“To groups”	30.0, (17.8–44.6)	22.0, (11.5–35.9)	26.0, (17.7–35.7)
“Room for improvement”	44.0, (29.9–58.7)	40.0, (26.4–54.8)	42.0, (32.2–52.3)
Total number of papers	50	50	100

## Papers which used laboratory rats

A similar search in Pubmed on “rat” and “experiment” found 483,490 papers. The first 50 of these with even identification numbers were published between 2015 and 2020. Four of them used mutant or genetically modified, the rest used wild-type rats. Twenty two of them involved experimental pathology, nineteen behaviour, seven physiology, one immunology and one pharmacology. Again, it was only the quality of the experimental design which was assessed, not the biological validity of results.

1. Nineteen (38 ± 0.3%) of the rat papers were placed in the “Design acceptable” category. Those involving behaviour were of notably high statistical quality (and complexity). Three stated that they had used the “Matched pairs” design and one had used a RB design without naming it. None of them mentioned either the CR or RB designs.
2. In eleven (22 ± 2.9%) papers, the rats were assigned to treatment groups. So it is unclear whether these papers had used the valid CR design or the in-valid RTTG design as discussed above for mice, although the latter seems to be more likely.
3. The “Room for improvement” group consisted of 20 (40 ± 4.0%) of the papers. These had not used the word “random” with respect to the assignment of treatments to subjects or vice versa and there was no evidence that they had used the RB, CR or other recognised experimental designs.

## Conclusions from the sample survey

Results for both mice and rats are summarised in Table 1. The quality of the experimental design in papers involving rats was slightly higher than that involving mice (Chi-sq. = 1.84, *p* = 0.04). This was largely due to the high quality of the behaviour (psychological) studies in the rat.

Combining the two species, 32 ± 4.7% of the papers were judged to have been designed and randomised to an acceptable standard, although none of them stated that they had used either the CR or RB design. One mouse paper had used a “Latin square” design. Another had used a “Completely randomised” design without naming it, and a mouse paper noted that “All experiments were performed independently at least three times.” Such repetition can lead to tricky statistical problems if results are to be combined^17^. Scientists wishing to build repeatability into their experiments could use the RB design, spreading the blocks over a period of time.

## Discussion and conclusions

Names matter. People, places, species and scientific procedures have names which can be used to identify and describe a subject or a procedure. Experimental designs also have names; “Completely randomised”(CR), “Randomised block”(RB), “Latin square”, “Matched pairs” etc. These can be found in textbooks which describe the characteristics and uses of each design^13^. However, none of the papers in the above survey mentioned either the CR or the RB design by name, although these are the only designs suitable for general use.

The widespread use of the statistically in-valid RTTG design, which is not found in any reputable textbooks, may account for a substantial fraction of the observed irreproducibility. Organisations which support pre-clinical research and training should ensure that their literature and web sites have been peer reviewed by qualified statisticians and that they refer to named, statistically valid experimental designs.

The RB and CR designs are quite versatile. They can be used for any number of treatments and sample sizes as well as for additional factors such as both sexes or several strains of animals, often without increasing the total numbers.

The first *clinical* trials were supervised by statisticians who adapted the CR design for such work. But scientists doing *pre-clinical* research have received little statistical support, so it is not surprising that so many of their experiments are incorrectly designed. High levels of irreproducibility are unlikely to be found in pre-clinical research in the pharmaceutical industry because the “PSI”, (the Association of Statisticians in the UK pharmaceutical industry), has about 800 members employed in the U.K.

Irreproducibility is wasteful and expensive. The employment of more applied statisticians in Academia to assist the scientists doing pre-clinical research would be an excellent investment.

## References

[1] Begley CG, Ellis LM (2012). Drug development: Raise standards for preclinical cancer research. Nature.

[2] Scott S, Kranz JE, Cole J, Lincecum JM, Thompson K, Kelly N, Bostrom A, Theodoss J, Al-Nakhala BM, Vieira FG, Ramasubbu J, Heywood JA (2008). Design, power, and interpretation of studies in the standard murine model of ALS. Amyotroph Lateral Scler.

[3] Freedman L.P., Cockburn IM, Simcoe TS: The Economics of Reproducibility in Preclinical Research. *PLoS Biol*; 13: e1002165.(2015). 10.1371/journal.pbio.1002165 PMC4461318 26057340

[4] Fiala C., Diamandis E.P.,: Benign and malignant scientific irreproducibility. *Clin Biochem*. May;55:1–2.(2018). 10.1016/j.clinbiochem.2018.03.015 29608889

[5] Boulbes D.R., Costello T., Baggerly K., Fan F., Wang R., Bhattacharya R., et al.: A Survey on Data Reproducibility and the Effect of Publication Process on the Ethical Reporting of Laboratory Research. *Clin Cancer Res* Jul 15;24(14):3447–55.(2018). 10.1158/1078-0432.CCR-18-0227 PMC6050098 29643062

[6] Marino M.,J.: How often should we expect to be wrong? Statistical power, P values, and the expected prevalence of false discoveries. Biochem Pharmacol May;151:226–33.(2018). 10.1016/j.bcp.2017.12.011 29248599

[7] Roberts I, Kwan I, Evans P, Haig S (2002). Does animal experimentation inform human healthcare? Observations from a systematic review of international animal experiments on fluid resuscitation. BMJ.

[8] Fisher RA (1960). The design of experiments.

[9] Montgomery DC (1984). Design and Analysis of Experiments.

[10] Festing MFW (1992). The scope for improving the design of laboratory animal experiments. Lab. Anim..

[11] Festing MF (2014). Randomized block experimental designs can increase the power and reproducibility of laboratory animal experiments. ILAR J..

[12] Festing MFW (2020). Experimental design and irreproducibility in pre-clinical research. Physiol. News.

[13] Festing MFW, Overend P, CortinaBorja M, Berdoy M (2016). The Design of Animal Experiments.

[14] Nevalainen T (2014). Animal husbandry and experimental design. ILAR J..

[15] Sprott RL (1967). Barometric pressure fluctuations: effect on the activity of laboratory mice. Science.

[16] Festing MFW (2006). Design and statistical methods in studies using animal models of development. ILAR J..

[17] Frommlet F, Heinze G (2020). Experimental replications in animal trials. Lab. Anim..

